# Separation of Magnetic Microparticles With Different Molecular Surface Functionalizations by Close‐to‐Surface Traveling‐Wave Magnetophoresis

**DOI:** 10.1002/smll.202512290

**Published:** 2026-02-09

**Authors:** Yahya Shubbak, Katharina Eichhorn, Nikolai Weidt, Arne Vereijken, Rico Huhnstock, Arno Ehresmann

**Affiliations:** ^1^ Institute of Physics and Center for Interdisciplinary Nanostructure Science and Technology (CINSaT) University of Kassel Kassel Germany; ^2^ Artificial Intelligence Methods for Experiment Design (AIM‐ED) Joint Lab of Helmholtzzentrum für Materialien und Energie, Berlin (HZB) and University of Kassel Berlin Germany

**Keywords:** dlvo‐interactions, lab on chip, magnetic domain engineering, magnetic field landscape, superparamagnetic particles

## Abstract

Magnetic microparticles (MPs) are at the core of a magnetic lab‐on‐a‐chip platform, where they can be used for liquid stirring, diffusion increase, and uptake, transport, concentration, and detection of analytes. A simple idea for analyte detection is to measure their change in magnetophoretic mobility upon analyte uptake. As typical biomolecular analytes are in the nanometer size range, they do not significantly increase the size of the MPs and, therefore, do not change their mobility away from any wall. Here, we show that MPs transported close to an underlying surface exhibit significantly different mobilities depending on their chemical surface properties. Specifically, traveling‐wave magnetophoresis leads to different average velocities for MPs with different molecular surface coverages despite having the same size and magnetic susceptibility. This effect is attributed to surface‐coverage‐dependent interactions between particle and substrate, mediated by the surrounding liquid, leading to different average distances between the substrate and MP. This, in turn, leads to different drag forces for their close‐to‐surface motion. We found that MPs of 2μm diameter covered by a polymer with carboxyl (COOH) end groups and a mixture of carboxyl and amino (${\mathrm{NH}}_{2}$) groups showed a large difference in their average close‐to‐substrate transport velocities in water at high driving frequency.

## Graphical abstract

Inducing surface‐near magnetic particle transport inside a static liquid via an engineered, dynamic potential energy landscape, different chemical surface properties of otherwise identical particles can be distinguished using simple optical observation. It potentially allows for quantitative in situ measurements of analyte‐binding to the particles' surfaces in lab‐on‐a‐chip applications by making use of their different electrostatic properties.

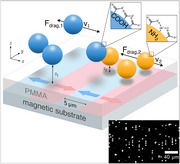

## 1 Introduction

Precise detection of low concentration analytes in body fluids (viruses [1, 2, 3], proteins [4], disease markers [5, 6, 7]) is often costly and time‐consuming, as in many cases, analyte amplification technologies (like polymerase chain reaction) are necessary. Alternatives without the need for amplification for quick and affordable medical diagnostics are, therefore, highly desired [8, 9]. Here, lab‐on‐a‐chip (LOC) devices are discussed as possible solutions [10, 11, 12, 13, 14, 15, 16], which are designed to integrate one or more laboratory functions into a small mm‐ to cm‐sized chip [17]. For such devices, controllably transported surface‐functionalized μm‐ or nm‐sized superparamagnetic particles (=magnetic particles, MPs) in a quiescent liquid [11, 12, 18, 19, 20] bear large application potential for a variety of tasks on chip. One possible key function is the detection of analytes in a sample or their concentration determination. In the past, this has been achieved by analyte‐bridge assays, where the analyte needs to bind to both a suitably functionalized MP surface at one binding site and at a second binding site to a fluorophore [21], another MP [18], or to the substrate [21]. In the first case, fluorescent analyte detection is addressed, in the second case, a change in mobility of transported MPs due to MP agglomeration, and in the third case, the immobilization of MPs, detected optically or by giant magnetoresistance (GMR) or tunnel magnetoresistance (TMR) sensors [22].

The idea of detecting disease markers upon binding to an MP surface by the associated change in MP mobility without the necessity of analyte bridges is intriguingly simple. Still, it has mainly been dismissed due to the large size difference between MPs (usually in the low μm range) and the disease marker (usually a protein in the nm range). For particles in free flow (i.e., far away from any wall), changes in the magnetophoretic mobilities cannot be quantified as the MP's hydrodynamic radius and, therefore, their viscous drag, will undergo only a tiny change upon binding, typically well below size variations of common MP batches.

Here, we show that for individual MPs with different molecular surface functionalizations, mobilities are very different when they are forced to move close to a surface. We explain this finding with the different liquid‐mediated particle‐substrate interactions for the different surface functional groups. The applied method and the current finding could be a major step toward a simple protocol to identify analyte binding to surface‐functionalized MP surfaces with large statistical significance without the need for bridge assays. Additionally, it sheds light on the impact of liquid‐mediated interactions between the MP surface and the underlying substrate surface on the MPs' motion dynamics. These interactions are crucial to understand when designing MP‐based LOC systems; however, to our knowledge, they have been widely disregarded so far. Reginka et al. [11] investigated the transport efficiency of protein‐functionalized MPs, highlighting the importance of the surrounding liquid medium and the electrostatic particle‐substrate interaction. Building on this, we have found that MP species can be distinguished based on this interaction.

In this work, MPs were transported over and close to a flat magnetic layer system with engineered in‐plane magnetic domains superposed by periodic magnetic field pulses, i.e., by traveling‐wave magnetophoresis [12, 23, 24]. Statistically significant differences in MPs' close‐to‐surface magnetophoretic mobilities have been determined for MPs with the same mean size and magnetic properties [25, 26] but different molecular surface functionalization.

### 1.1 Experiment and Method

#### 1.1.1 Magnetic Microfluidic Chip and Experiment

**Figure 1:**
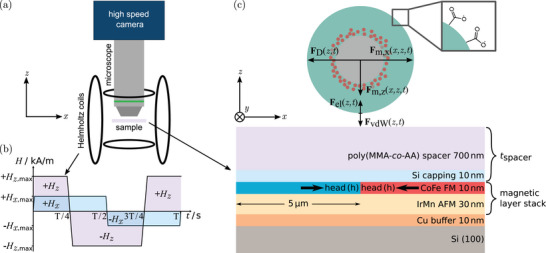
(a) Experimental setup. (b) Periodically switching trapezoidal field pulses $H_{\mathrm{ext}}$ with its components in z‐ and x‐directions. (c) Acting forces, MPs, magnetic layer system, and coordinate system. MPs had a copolymer core (grey), and a magnetite nanoparticle shell (red‐brown) covered by an additional polymer coating (green shell). Surface functionalization is sketched for COOH that partially deprotonates in water [31]. x=0 of the coordinate system is at the center of a hh‐DW, z=0 is the interface of the spacer layers (total thickness $t_{\mathrm{spacer}}\approx 700\;\mathrm{nm}$) to the liquid. For t=0 in (b), the MP is located above a hh‐DW. The two components of the magnetic force $F_{m}\left(x,z,t\right)$ are time‐dependent due to the external field pulses. The drag force $F_{D}\left(z,t\right)$ is opposing and proportional to the velocity vector of the MP. Liquid‐mediated electrostatic $F_{\mathrm{el}}\left(z,t\right)$ and van der Waals $F_{\mathrm{vdW}}\left(z,t\right)$ forces are mainly acting along the z direction. Layer thicknesses displayed are not to scale.

The substrate was a silicon wafer covered by a magnetic exchange‐bias (EB) layer system (Figure 1c). The layer system has been patterned into parallel‐stripe magnetic domains with head‐to‐head (hh)/ tail‐to‐tail (tt) in‐plane magnetizations in adjacent stripes by ion bombardment induced magnetic patterning (IBMP) [27, 28, 29] of EB layers without modifying the flat surface topography [12]. The domains are separated by domain walls (DW). Bombardment has been performed using a home‐built Penning ion source [30] through a lithographic resist mask of about 600nm thickness with a dose of $2.5\times 10^{15}\;{\mathrm{cm}}^{-2}\;10\;\mathrm{keV}$ He $\mathrm{ions}\;{\mathrm{cm}}^{-2}$ in an in‐plane magnetic field of 80 mT opposing the initially set EB direction and subsequent mask removal. A microfluidic chamber was fabricated by a Parafilm sheet with an opening of about 8 mm × 8 mm adhered to the substrate. Suspensions of MPs have been pipetted into the Parafilm window, which was then sealed with a cover slip.

#### 1.1.2 Acting Forces

Figure 1c sketches the relevant forces from the substrate's surface acting on the MPs. The latter are exemplarily located above a hh‐DW center originating from the underlying magnetic layers. These layers cause the acting magnetic forces. The lateral directed motion is driven by the magnetic gradient force $F_{m},x\left(x,z,t\right)=V\left(\chi_{\mathrm{MP}}-\chi_{\mathrm{fl}}\right)\mu_{0}{\left(\left(H_{\mathrm{eff}}\nabla \right)H_{\mathrm{eff}}\right)}_{x}$ on an MP, where V is the MP's volume, $\chi_{\mathrm{MP}}$ is the particle's magnetic susceptibility, $\chi_{\mathrm{fl}}$ the susceptibility of the fluid, $\mu_{0}$ is the vacuum permeability, and $H_{\mathrm{eff}}$ the effective field (sum of static stray field and superposed external fields) at the position of the particle. The opposing drag force $F_{D}\left(z,t\right)$ increases with increasing instantaneous velocity and is described for low Reynold's numbers by [32]

(1) $$F_{D}\left(z,t\right)=-6\pi r\eta \frac{dx}{dt}f_{D}\left(z\right)$$

where r is the MP's radius, η is the liquid's viscosity ($1.0093\times 10^{-3}\;\mathrm{Pa}s$ at T=293.15K for water [12]), and $f_{D}\left(z\right)$ is a z‐height dependent drag coefficient [32]:

(2) $$\begin{array}{ccc} f_{D}\left(z\right) & = & \left[1-\frac{9}{16}\left(\frac{r}{r+z}\right)+\frac{1}{8}{\left(\frac{r}{r+z}\right)}^{3}\right.\\ & & {\left.-\frac{45}{256}{\left(\frac{r}{r+z}\right)}^{4}-\frac{1}{16}{\left(\frac{r}{r+z}\right)}^{5}\right]}^{-1} \end{array}$$

**Figure 2:**
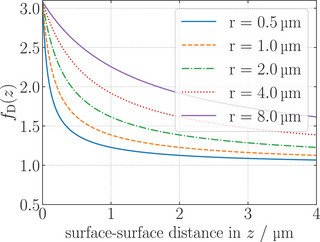
Drag coefficient as a function of distance z between substrate surface and surface of MP facing the substrate. The orange dashed line (r=1μm) shows the radius of the MPs used in our study.

Here, z is the closest surface‐to‐surface distance between MP and substrate [32]. $f_{D}\left(z\right)$ decays strongly from the substrate's surface into the liquid. The drag coefficient as a function of distance z to the substrate surface is plotted in Figure 2 for different MP radii, showing that there is a relevant change in drag in the MP‐to‐surface distance regime of a few μm, where the MP transport takes place [11, 33]. Therefore, the varying drag for different MP‐surface distances will influence the MP motion and their average velocity.

We will now briefly describe the liquid‐mediated interactions between MP and substrate surfaces. For that, the zeta potentials $\zeta_{\mathrm{MP}}$ and $\zeta_{s}$ of MP and substrate surfaces are commonly used as approximations for the potentials caused by surface charges [11, 12, 32]. The electrostatic force between particle and substrate can then be written as

(3) $$\begin{array}{ccc} F_{\mathrm{el}}\left(z,t\right) & = & \frac{2\pi \varepsilon_{0}\varepsilon_{r}r\kappa }{1-\exp\left(-2\kappa z\right)}\left[2\zeta_{\mathrm{MP}}\zeta_{s}\exp\left(-\kappa z\right)\right.\\ & & \left.\;\pm \;(\zeta_{\mathrm{MP}}^{2}+\zeta_{s}^{2})\exp\left(-2\kappa z\right)\right]e_{z} \end{array}$$

where $\varepsilon_{0}\varepsilon_{r}$ is the permittivity of the liquid medium, and κ is the Debye length

(4) $$\kappa =\sqrt{\frac{2000N_{A}e^{2}I}{\varepsilon_{0}\varepsilon_{r}k_{B}T}}$$

with the Avogadro constant $N_{A}$, the elementary charge e, the ionic strength of the electrolyte I, the Boltzmann constant $k_{B}$ and temperature T [11, 32].

The electrodynamic van der Waals force between two materials interacting in a medium can be approximated by the Hamaker theory [32], resulting in:

(5) $$F_{\mathrm{vdW}}\left(z,t\right)=-\frac{A_{132}r}{6z^{2}}\left[\frac{1}{1+14z/\lambda_{\mathrm{ret}}}\right]e_{z}$$

where $A_{132}$ is the Hamaker constant for a material 1 interacting with material 2 in a medium of material 3. Additionally, the effect of retardation $\lambda_{\mathrm{ret}}$ is taken into account, which is a correction factor for the acting force when the distance between the surfaces exceeds 5nm [34]. Both interactions, electrostatic and electrodynamic, have in the past been summarized as Derjaguin, Landau, Verwey, and Overbeck (DLVO) forces [32, 35, 36].

In the present case, MPs are coated by an acrylic acid derivative polymer that possesses −COOH groups at their surface, which are partially deprotonated to carboxylate anions $-{\mathrm{COO}}^{-}$ in water [31]. This leads to negative surface charges and, therefore, to a negative $\zeta_{\mathrm{MP}}$‐potential [32]. Similarly, negative charges are on the poly‐(methyl methacrylate‐*co*‐acrylic acid) [P(MMA‐*co*‐AA)]‐spacer surface, leading to a negative $\zeta_{s}$ ($\zeta_{s}=-45\;mV$ at pH=7 [37]). For zeta potentials of MP and substrate being both negative, the DLVO forces are repulsive.

Adding ${\mathrm{NH}}_{2}$‐groups to the surface of the polymer will induce positive charges at the MP‐surface because partial protonation to ${\mathrm{NH}}_{3}^{+}$ occurs in water [31]. The positive charges will compensate for some of the negative charges of the acrylic acid surface to which the ${\mathrm{NH}}_{2}$‐functional groups are bound. This decreases the absolute magnitude of the $\zeta_{\mathrm{MP}}$‐potential but maintains its negative sign. For the following discussion, MPs with the mixture of COOH and ${\mathrm{NH}}_{2}$ at their surfaces will be named ${\mathrm{NH}}_{2}$‐MPs. Using a zetasizer (Zetasizer Nano ZS90, Malvern) the zeta‐potentials of the MP suspensions in double distilled water(${\mathrm{ddH}}_{2}O$) of −45mV for MPs with only ${\mathrm{COO}}^{-}$ functionalized surfaces and −35mV for the ${\mathrm{NH}}_{3}^{+}$ functionalized surface have been determined at pH=7 and room temperature (RT). The overall negative $\zeta_{\mathrm{MP}}$ ensures repulsive interaction between MP and substrate surface, avoiding adhesion to the surface and thus immobilization of the particles.

#### 1.1.3 Particle Transport Mechanism: Traveling‐Wave Magnetophoresis

The periodic magnetic field landscape (MFL) above the parallel‐stripe in‐plane magnetized domain pattern (stripe‐domain width = 5μm, periodicity d=10μm) (Figure 1c) leads to a static periodic potential energy landscape for the MPs creating a magnetic force component $F_{m,z}\left(x,z\right)$ toward the substrate [38]. This force ensures a lateral transport in which close‐to‐substrate positions of the MPs are maintained. Similar to previous works [11, 38, 39, 40], a trapezoidal magnetic field pulse sequence (Figure 1b) has been superposed on the static MFL, leading to a stepwise hopping motion [39] with a defined lateral direction. Due to the used parallel‐stripe domains, no field gradient exists in the y‐direction, ensuring no magnetic forces act in that direction. A transport from one DW to the neighboring consists of the following steps (see Figure 1b, [24]): At t=0 ($H_{x}\left(x=0,t\right)=0,H_{z}\left(x=0,t\right)=+H_{z,\max}$) an external trapezoidal magnetic field pulse in positive z‐direction is in its plateau, which lifts the degeneracy of the MP's potential energy minima over each other DW [12, 38]. When no x‐field is applied, motion of MPs residing on potential energy maxima occurs statistically in ±x‐directions toward the two closest potential energy minima, collecting all MPs over every other DW. Adding a field in the +x‐direction shifts the potential‐energy minima from the DW centers slightly toward the +x‐direction, with MPs following this shift. At t=T/4 the z‐field is reversed while the x‐field is at its plateau, shifting the potential energy minima close to the neighboring DW centers with particles accelerating toward +x up to a steady‐state velocity and then decelerating again when the next potential‐energy minimum is reached. z‐ and x‐fields then periodically switch signs, causing a stepwise transport of the MPs with the period length of the pattern traveled after T of the external field sequence. For the following discussion we define the frequency of the driving field pulses as ν=1/T.

#### 1.1.4 Transport Velocities

Apart from the instantaneous MP velocity, which changes with time, and the steady‐state velocity, which results from the equilibrium between drag and driving forces (assuming a constant driving force), the most important observable in the current context is the MP velocity averaged over the whole experiment. An analytical expression for this average velocity has been formulated as [23]

(6) $$v:=\langle v\rangle =\left\{\begin{array}{cc} \nu d, & \nu \leq \nu_{c}\left(z\right)\\ (\nu -\sqrt{\nu^{2}-\nu_{c}^{2}})d, & \nu >\nu_{c}\left(z\right) \end{array}\right.$$

with

(7) $$\nu_{c}\left(z\right)=\frac{\bar{\chi }\;\mu_{0}\;\sigma_{0}\;H_{\mathrm{eff}}}{36\pi \;\eta \;f_{D}\left(z\right)}{\left(2\pi \beta \right)}^{2}\exp\left(-2\pi \beta \right)$$

where $\bar{\chi }$ is the shape corrected bead susceptibility, $\sigma_{0}$ denotes the effective magnetic pole distribution within the underlying substrate, and β=r/d is the ratio between MP radius r and the domain periodicity d, and $H_{\mathrm{eff}}$ is the effective field, being the vector sum of the substrate's stray field and the external field. Equation (6) describes two distinct velocity regimes, a regime where the average velocity linearly increases with the frequency of the field pulses, independent of the MP, and a nonlinear regime, where the MPs are not able to completely follow the quickly changing field pulses, resulting in a decreasing average velocity with increasing driving frequency. Both regimes are separated by the critical frequency $\nu_{c}$. Extending the previous analysis [23] by the z‐dependent drag force for close‐to‐surface motion, the critical frequency therefore depends on the MP's distance to the surface as can be seen in Equation (7) where the drag force coefficient $f_{D}\left(z\right)$ has been included. This opens the possibility that not only MPs of different sizes or loaded with macroscopic cargo may be separated by different critical frequencies [23], but also MPs with different liquid‐mediated MP‐substrate interactions due to a different surface coverage by small functional molecular groups or analytes. Differing surface coverages may lead to different average distances between MPs and the surface, ultimately leading to a modified average velocity. In the following, we will show that this concept is experimentally feasible.

### 1.2 Experimental Results

#### 1.2.1 MP Transport and Tracking

**Figure 3:**
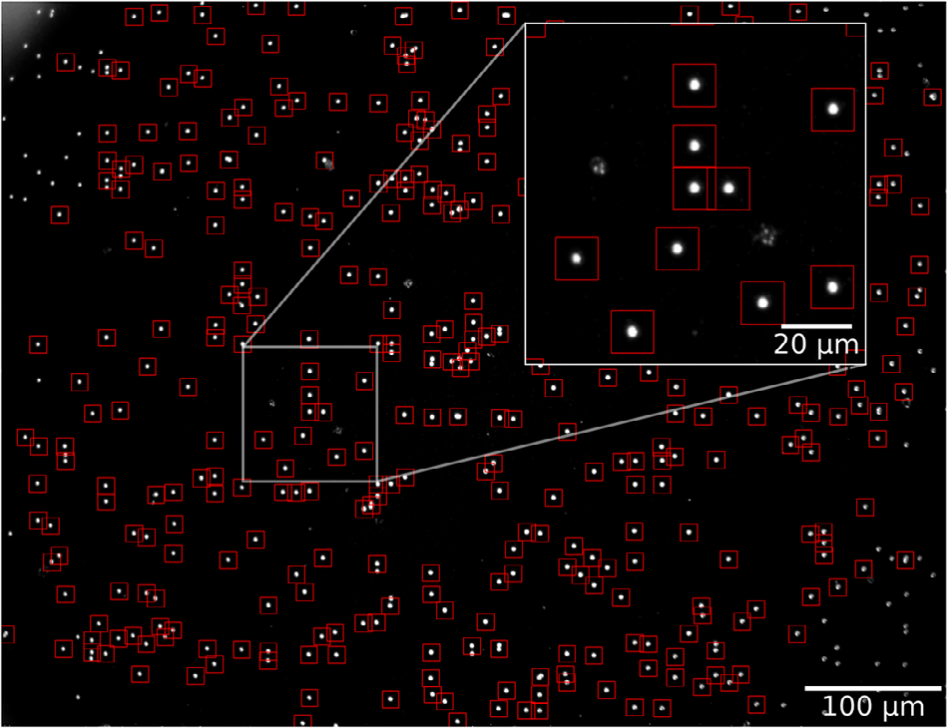
Exemplary video frame of a dark‐field mode recording for a transport experiment of COOH‐functionalized MPs. MPs recognized by the automated tracking algorithm are marked with red squares. The inset of a zoomed frame area shows that static artifacts on the substrate surface are correctly ignored.

**Figure 4:**
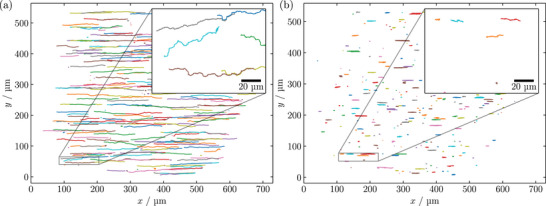
MP trajectories extracted from video recordings of two MP transport experiments with the same number of transport steps. (a) At ν=2.5Hz MPs show directed transport along the x‐direction. The inset shows magnified trajectories, displaying the superposition of a directed transport in +x‐direction with Brownian motion in y‐direction. (b) At ν=25Hz ($\nu >\nu_{c}$) MPs show directed transport in +x‐direction with shorter trajectories as back‐slippages become frequent. This can lead to oscillatory motion around a DW (point‐like trajectory).

The following transport experiments were conducted using micromer‐M MPs with d=2.0+−0.4μm nominal diameter, magnetite content varying from 2.2%to4.0%, and COOH or COOH‐${\mathrm{NH}}_{2}$‐covered surfaces, as stated by the manufacturer. We have verified, to the best of our ability, the structural, surface, and magnetic properties of the used MPs, primarily through the magnetophoretic mobility study presented in the Supporting Information. They consist of nanoscopic magnetite grains dispersed in a copolymer matrix covered by an additional polymer shell, enabling the investigated surface functionalization (see sketch of Figure 1c). Due to the dispersed magnetite nanoparticles, the MPs are superparamagnetic. Applied magnetic field amplitudes of the trapezoidal pulses were $\mu_{0}H=2.3\mathrm{mT}$ in x− and $\mu_{0}H=2.6\mathrm{mT}$ in z‐directions, with pulse frequencies ν between 0.5Hzto25Hz. The linear rise time of the trapezoidal field pulse applied to the Helmholtz coils (see methods) is three orders of magnitude slower than the acceleration time of MPs in our case (ca. 1μs) [24]. The magnetic field alteration rate was 4 $\times 10^{3}$ mT $s^{-1}$. MP transport videos of 5s length have been recorded at a video frame rate of 1000 fps through an optical reflected‐light microscope at 20× magnification. The dark‐field microscopy mode was used to optimize the visibility of the MPs for the tracking procedure. An exemplary frame of a recording with a magnetic field pulse period length of T/4=100ms (ν=2.5Hz) can be seen in Figure 3. Individual MPs are visible as bright spots against a black background. MPs detected by the tracking algorithm are marked with red squares. A map of MP positions over time is displayed in Figure 4, here for COOH MPs, displaying their trajectories at (a) ν=2.5Hz and (b) ν=25Hz for the same amount of steps for better comparison. It is immediately evident that MPs at field‐pulse frequencies in the linear transport regime (a) are traversing larger distances than at frequencies in the nonlinear regime (b).

#### 1.2.2 Average Velocity

**Figure 5:**
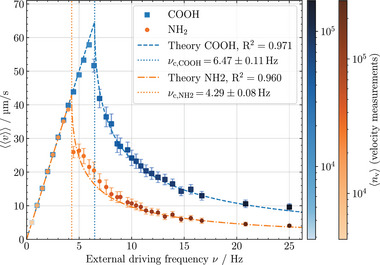
Average MP transport velocities $\langle \langle v\rangle \rangle \left(\nu \right)$ for the two differently surface‐functionalized MP types averaged over the number of tracked MPs as a function of the external field pulse frequency ν. The one‐parameter fits to the theoretical model (see Equation 6) are shown by dashed (COOH‐functionalization) and dashed‐dotted (${\mathrm{NH}}_{2}$‐functionalization) lines, respectively. The critical frequencies determined by the fit are marked by dotted vertical lines. The number of tracked MPs ranges between 110 and 340 for COOH and 210 and 540 for ${\mathrm{NH}}_{2}$. The color‐map shows how many velocity measurements $n_{v}$ are included in each $\langle \langle v\rangle \rangle$ data point. Here, the number of transport steps (=velocity measurements) increases with increasing ν related to the transport concept (see Figure 1b) with $n_{\mathrm{steps}}=4\;\nu t$.

The experimentally determined average transport velocities $\langle v\rangle$ of individual MPs have further been averaged over the number $n_{\mathrm{MP}}$ of tracked MPs (between 110 and 540 MPs), yielding $\langle \langle v\rangle \rangle$. This quantity is displayed in Figure 5 for MPs with the two different surface functionalizations (COOH and ${\mathrm{NH}}_{2}$) as functions of the driving frequency ν. For each frequency and particle type, one video of 5000 frames was recorded. However, the averaging of $\langle v\rangle$ over the number of all tracked MPs for a given ν results in large statistical significance.

The two transport regimes are clearly displayed: At low frequencies $\nu <\nu_{c}$, MPs follow the stepwise relocation of their potential energy minima in the phase‐locked linear transport regime. The increase in the driving frequency linearly increases $\langle \langle v\rangle \rangle$. In this regime, the averaged velocity at a given driving frequency and the increase with frequency are independent of the MP type, including its surface functionalization, as predicted by Equation (6).

The critical frequencies (Equation (7)) for the two differently surface‐functionalized MP types are largely different, corroborating the possibility of distinguishing them by their average transport velocities in the nonlinear transport regime us traveling‐wave magnetophoresis. The critical frequencies of the measured $\langle \langle v\rangle \rangle \left(\nu \right)$ have been determined by a one‐parameter fit of the experimental data to Equation (6). This allowed for an accurate determination of the critical frequencies, which were found to be $\nu_{c,\mathrm{COOH}}=6.47\pm 0.11\;\mathrm{Hz}$ and $\nu_{c},{\mathrm{NH}}_{2}=4.29\pm 0.08\;\mathrm{Hz}$, with uncertainties given for a 95% confidence level).

As a control, we performed a separate magnetophoretic velocity measurement far away from any surface to verify that the two differently surface‐functionalized MP batches used are, in fact, identical in their magnetophoretic behavior in free fluid, as described in the Supporting Information. We found the same velocities for both MP types, confirming that the close‐to‐surface transport is key. The susceptibility of the MPs is found to be $\chi_{\mathrm{COOH}}=0.26\pm 0.01$ and $\chi_{{\mathrm{NH}}_{2}}=0.27\pm 0.01$, and are indeed the same as claimed by the manufacturer.

### 1.3 Discussion

The average traveling‐wave magnetophoretic velocities in their nonlinear transport regimes (Equation (6)) for two differently surface‐functionalized MP batches differ significantly when transported close to a surface. Different molecular surface functionalizations, though only negligibly changing the radius of the MP, induce different interactions with a nearby surface. Here, especially the repulsive components of the DLVO forces are different, leading, together with the attractive magnetic force (being equal for both MP batches), to different average equilibrium heights of the MP over the surface. In the present case the COOH‐functionalized MPs with a more negative $\zeta_{\mathrm{MP}}$ potential (−45mV compared to −35mV for the ${\mathrm{NH}}_{2}$‐functionalized MPs), are repelled stronger by the polymer spacer, which also has a negative zeta‐potential ($\zeta_{s}=-45\;\mathrm{mV}$). The higher electrostatic repulsion leads to a larger average equilibrium distance, which in turn decreases the drag force close to the surface, described by the height‐dependent drag coefficient $f_{D}\left(z\right)$. A lower drag coefficient then leads to a higher critical frequency and quantifiably higher average velocities, despite the large variation of MP diameters and magnetic content within a single batch. A simple control experiment, in which MPs were transported in free flow, confirmed that the observed distinction comes from close‐to‐surface transport, as MPs exhibit the same velocity in a magnetic gradient field regardless of their surface functionalization (see Supporting Information).

Two facts are not yet included in the simple model described above: (1) The magnetic field of the substrate's domain pattern decreases with increasing distance to the ferromagnetic layer [41], leading to smaller induced magnetic moments of the MPs and slight modifications in the potential landscape. This counteracts in part the effect of decreasing drag with increasing distance to the surface. As the distance dependence of the stray field of the parallel‐stripe domains pattern is weaker than that of the friction coefficient, the net effect remains. (2) Trajectories for MPs transported by the present traveling‐wave magnetophoresis are inherently 3D [39]. For the derivation of Equation (6), this fact has been neglected and needs to be included in a refined theory, which is beyond the scope of the present work.

Finally, we may consider investigating different particle sizes in the future studies to probe the limits of this method for potential analyte detection. As can be seen in Figure 2, the smaller the particle diameter, the more sensitive the change of the drag coefficient with varying substrate distance (in a regime close to the substrate surface). However, even though MPs in the sub‐micrometer size regime are commercially available, at some point their size will be below the Abbe limit of the used microscope, and the MP cannot be imaged optically. The smaller size also comes with the trade‐off of a smaller surface, limiting the analyte surface coverage that leads to the intended modification of the particle‐substrate interaction. Larger MPs (above 5μm diameter), on the other hand, will experience a superposition of opposing stray field directions in their volume, emerging from neighboring domain walls, as the width of a stripe domain is smaller than the MP's diameter.

## 2 Conclusion

This study presents a quantitative analysis of MP transport near a substrate surface in a static liquid environment by traveling‐wave magnetophoresis, enabling the separation of MPs only different in their chemical surface properties. Using optical dark‐field microscopy and automated single‐particle tracking, we observed significant differences in the average velocities between COOH‐ and ${\mathrm{NH}}_{2}$‐functionalized MPs as a function of the external driving field frequency. The difference in transport velocities is explained by a height‐dependent drag coefficient, whereby height differences originate from different liquid‐mediated DLVO forces acting between substrate and MP. Consequently, the separation distance between MP and substrate surface is different for the two MP types, leading to a modified drag force during lateral motion. The observed dependence of $\langle \langle v\rangle \rangle$ on the driving frequency is in good agreement with the underlying theory.

Our results are the basis for sorting/separating MPs solely based on their surface characteristics and pave the way for molecular‐analyte detection relying entirely on a comparably simple technical approach. Being able to move hundreds of MPs in parallel opens up new possibilities to create parallelized analyte detection devices. Large individual variations in MP properties of a batch, which are unavoidable in the MP fabrication process, did not influence the clear outcome of our experiment.

## 3 Materials and Methods

### 3.1 Fabrication of Magnetically Patterned Substrate

The prototypical EB magnetic stripe pattern was fabricated by IBMP [27, 28, 29] of a ${\mathrm{Cu}}^{10\;\mathrm{nm}}/$ ${\mathrm{Ir}}_{17}{\mathrm{Mn}}_{83}^{30\;\mathrm{nm}}/{\mathrm{Co}}_{70}{\mathrm{Fe}}_{30}^{10\mathrm{nm}}/{\mathrm{Si}}^{10\mathrm{nm}}$ layer system deposited onto naturally oxidized Si (100). The layer system was created by RF sputter deposition (Leybold Heraeus Z400) at RT with a magnetic field of 35 mT applied to initialize the in‐plane direction of the EB. It was then field cooled to further strengthen the in‐plane EB through annealing in a vacuum chamber at $300^{\circ }C$ for 60 min in a magnetic field of 150mT, aligned parallel to the initial EB, and then cooling down to RT. The masked sample was then bombarded with helium ions at a kinetic energy of 10keV, using a home‐built Penning ion source [27, 28, 29]. The ion dose was $2.5\times 10^{15}\;{\mathrm{cm}}^{-2}$. Simultaneously, a homogenous magnetic field of 80mT, antiparallel to the direction of the initialization field, was applied during the ion bombardment to create a hh/tt magnetic domain configuration (see Figure 1). After the ion bombardment, the photoresist was removed by treating the sample for 2 h with TechniStrip NI555 solution at $80^{\circ }C$, followed by ultrasonication for 3 min at RT. The sample was then cleaned with acetone and isopropanol and dried in a stream of nitrogen. Finally, the sample was spin‐coated with 700nm copolymer poly(methyl methacrylate‐*co*‐acrylic acid), resulting in a total spacer layer thickness $t_{\mathrm{spacer}}$ of 710nm above the magnetic layer stack. The polymer solution used was the E‐Beam Resist AR‐P 617.06 from ALLRESIST GmbH. It was soft‐baked at $100^{\circ }C$ for 10 min.

### 3.2 MP Transport

The underlying transport concept of the experiments is based on previous studies and can be summarized as follows [24, 38, 42]: The magnetic particles micromer‐M with a diameter of 2μm purchased from micromod Partikeltechnologie GmbH with either COOH‐ or ${\mathrm{NH}}_{2}$‐functionalization were diluted in ${\mathrm{ddH}}_{2}O$ from the stock solution to a concentration of $8\times 10^{6}$ $\text{MPs}\cdot {\text{mL}}^{-1}$. They have a magnetization of $4.8\;A\;m^{2}\;{\mathrm{kg}}^{-1}$ with a saturation magnetization of $>6.5\;A\;m^{2}\;{\mathrm{kg}}^{-1}$, and a density of $1.1\;g{\mathrm{cm}}^{-3}$ [25, 26]. 10μL of diluted MP solution was then pipetted on top of the 1.5cm times 1.5cm magnetically patterned substrate with a Parafilm window to support a cover slide, effectively creating a simple microfluidic chamber, allowing for experimentation with minimum evaporation. It is placed in the center of a Helmholtz coil set‐up with magnetic fields in x and z directions. Pulsed magnetic fields with amplitudes of 2.3mT in the x− and 2.6 mT in the z‐direction were applied. The study consists of capturing the particle's motion depending on the external driving frequency of the pulsed external fields using a highspeed camera with a 20× magnification objective of a reflection light microscope (Zeiss Axiotech D). The linear rise time of the trapezoidal field pulse applied to the Helmholtz coils from $H_{x}=-2.3\mathrm{mT}\;\;(H_{z}=-2.6\mathrm{mT}$) to $H_{x},\max=2.3\mathrm{mT}\;\;(H_{z},\max=2.6\mathrm{mT}$) was $\Delta t_{r},x=1144\;\mu s\;\;\left(\Delta t_{r},z=1294\;\mu s\right)$ [24]. The camera recorded images at a framerate of 1000 fps and thus allows in situ observation of the MP movement with ms resolution. During the 5s capture time, the particles were moving back and forth up to 40 times (details: see methods). The reason for alternating sequences was to observe and analyze the same MPs moving within the microscope's field of view. The experiment was then repeated with a changed frequency. After the first series of experiments for the first MP species, the substrate was rinsed with ${\mathrm{ddH}}_{2}O$, and the experimental series was repeated for the second species at roughly the same substrate position.

### 3.3 MP Tracking and Statistical Analysis

The acquired image sequence of 5000 frames per experiment is cropped from 1920×1080 pixels to 1408×1080 pixels to avoid inhomogeneous illumination at the field of view fringes. MPs located in this region of interest are then tracked using the Python [43] package trackpy [44] 0.6.2 with Python 3.12. For that, any features, i.e., particles, with these three parameters are found: diameter = 13, which is the size of particles in pixels, minmass = 1000, a measure of the minimum integrated brightness against a (dark) background, and separation = diameter+1, chosen to ensure no overlapping of particles. Other tracking parameters are left at the default values. The resulting DataFrame contains, among other information, x‐ and y‐position for each tracked, uniquely numbered particle for all given video frames, which are then used to determine the trajectories and the velocity. For that, a series of data analysis steps is done. First, grouping of the DataFrame by particle makes x‐ and y‐coordinates a coherent time series for each particle, lending them actual trajectories. Trajectories shorter than 2000 frames (two‐fifths of the total recorded frames) are discarded, as they tend to be spurious, as further described in the Supporting Information. This happens when the algorithm breaks the linking for a given particle and continues as if it were a new particle, which has a negative bias on $\langle \langle v\rangle \rangle$. However, true trajectories are also filtered out if they are just transported outside the field of view while presenting fewer than 2000 linked frames or, likewise, just entering the frame. Additionally, trajectories shorter than 20μm length were removed in the linear regime, which is also further described in the Supporting Information. Then, the velocity of a given trajectory is calculated by taking the derivative of the x position with respect to time. Afterward, MP velocity outliers were removed using the interquartile range (IQR) method. Error bars in Figure 5 represent a confidence level of 95% calculated using the standard error of the mean (SEM) multiplied by the critical value Z=1.96 for large n, where $\mathrm{SEM}=\sigma /\sqrt{n}$, whith σ being the sample standard deviation and n being the sample size.

The sample size n for each experiment ranges between 110 and 340 for COOH and 210 and 540 for ${\mathrm{NH}}_{2}$. Multiplying n with the number of transport steps the MP took results in the number of velocity measurements $n_{v}$ taken per experiment for the given $\langle \langle v\rangle \rangle$. This is color‐coded in Figure 5 and ranges between $n_{v}=2900$ to $n_{v}=170000$ for COOH and between $n_{v}=4500$ to $n_{v}=270000$ for ${\mathrm{NH}}_{2}$. Note that the number of velocity measurements is naturally increasing with larger ν due to the MP performing more transport steps in the fixed video recording duration.

## Conflicts of Interest

The authors declare no conflicts of interest.

## Supporting information


**Supporting File 1**:smll72782‐sup‐0001‐SuppMat.pdf.

**Supporting File 2**:admi202300226‐sup‐0002‐VideoS1.mp4.

## Data Availability

The data is openly available in figshare with the following DOI: https://doi.org/10.6084/m9.figshare.30295648.
