# Supplementary material for: Separation of Magnetic Microparticles With Different Molecular Surface Functionalizations by Close‐to‐Surface Traveling‐Wave Magnetophoresis
Source: Small. 2026 Feb 9;22(21):e12290. doi: 10.1002/smll.202512290 (PMC13081117; doi:10.1002/smll.202512290)
Supplement: Supplementary file 1 — Supporting File 1:smll72782‐sup‐0001‐SuppMat.pdf. [file SMLL-22-e12290-s001.pdf]

Separation of Magnetic Microparticles with Different Molecular Surface Functionalizations by Close-to-Surface Traveling-Wave Magnetophoresis

Yahya Shubbak Katharina Eichhorn Nikolai Weidt Arne Vereijken Rico Huhnstock Arno Ehresmann**

Y. Shubbak*[1,2], K. Eichhorn[1], N. Weidt[1,2], A. Vereijken[1,2], Dr. R. Huhnstock[1,2], and Prof. A. Ehresmann**[1,2]
Email Address:
*y.shubbak@physik.uni-kassel.de
**ehresmann@physik.uni-kassel.de
[1]Institute of Physics and Center for Interdisciplinary Nanostructure Science and Technology (CINSaT), University of Kassel, Heinrich-Plett-Strasse 40, 34132 Kassel, Germany.
[2]Artificial Intelligence Methods for Experiment Design (AIM-ED), Joint Lab of Helmholtzzentrum für Materialien und Energie, Berlin (HZB) and University of Kassel, Hahn-Meitner-Platz 1, 14109 Berlin, Germany

Keywords: *Lab on Chip, DLVO-interactions, Magnetic Domain Engineering, Magnetic Field Landscape, Superparamagnetic Particles*

Supporting Information

Video of MP Motion

Real-time video recording of exemplary MP transport at $\nu = 2.5\,\text{Hz}$ for the surface functionalization NH_2 is provided in the online version.

Filtering Spurious Trajectories

Trajectories of > 2000 frames duration were discarded. The tracking algorithm is not well-suited for tracking dense particle conglomerates. The large number of particles required for good statistics thus comes at the price of dismissing a certain number of trajectories. This is because they are tracked and lost when two or more particles come into proximity. This creates patchy trajectories with missing information. To illustrate this behavior, two trajectory maps are shown: one with no filtering for spurious trajectories (**Figure S1**) and another with filtering of 2000 Frames (i.e., trajectories are at least 2000 frames long) (**Figure S2**). As visible in Figure S2, even after filtering, trajectories that appear short remain. These are trajectories that are present for at least 2000 frames, but only oscillate around a DW. Two types of MPs will create this behavior: MPs experiencing slips back or MPs stuck either on a surface defect or an immobile particle. The former is to be expected in the non-linear regime and must be kept. The latter, of course, are MPs that have to be filtered out. In the linear regime, this is simply done by removing MPs that haven't traveled at least 20 µm, the result of which can be seen in **Figure S3**. In the non-linear regime, filtering these MPs out would significantly alter the outcome in a non-physical way (see Equation 6 in the Manuscript); therefore, they are kept.

Number of MPs

The number of tracked MPs n_{MP} varies, after filtering for spurious particles, between 110 and 340 for COOH and 210 and 540 for NH_2-functionalized MPs and can be seen for each individual experiment in **Figure S4**. The different number of MPs within the same MP type is caused by MPs constantly moving into and out of the microscope's field of view. Within the linear regime, more MPs can do so for larger ν. The difference among the different MP species is because the concentration of NH_2-MPs was larger than stated by the manufacturer.

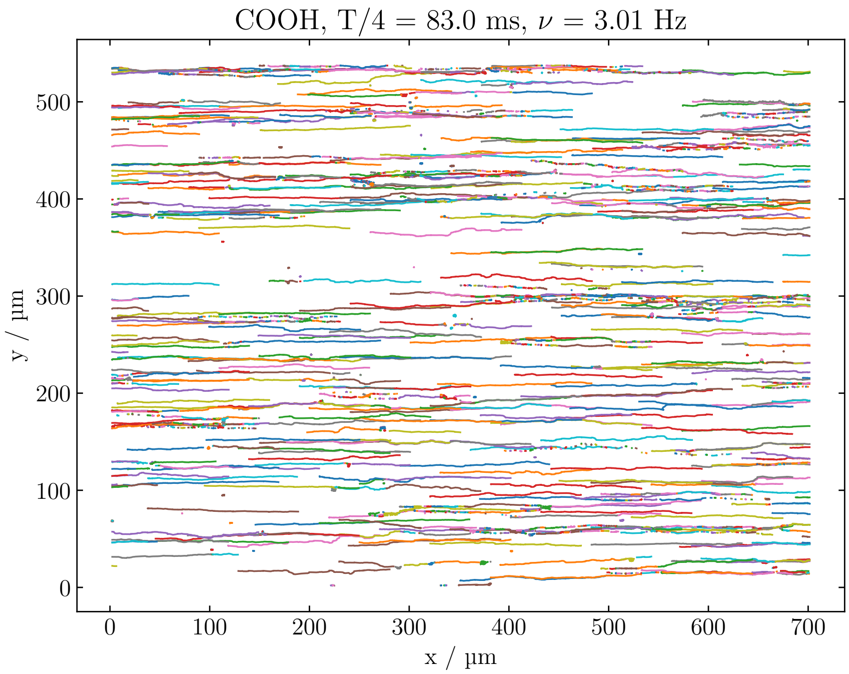

Figure S1: Trajectory map of COOH MPs at $\nu = 3.01\,\text{Hz}$, no filtering applied.

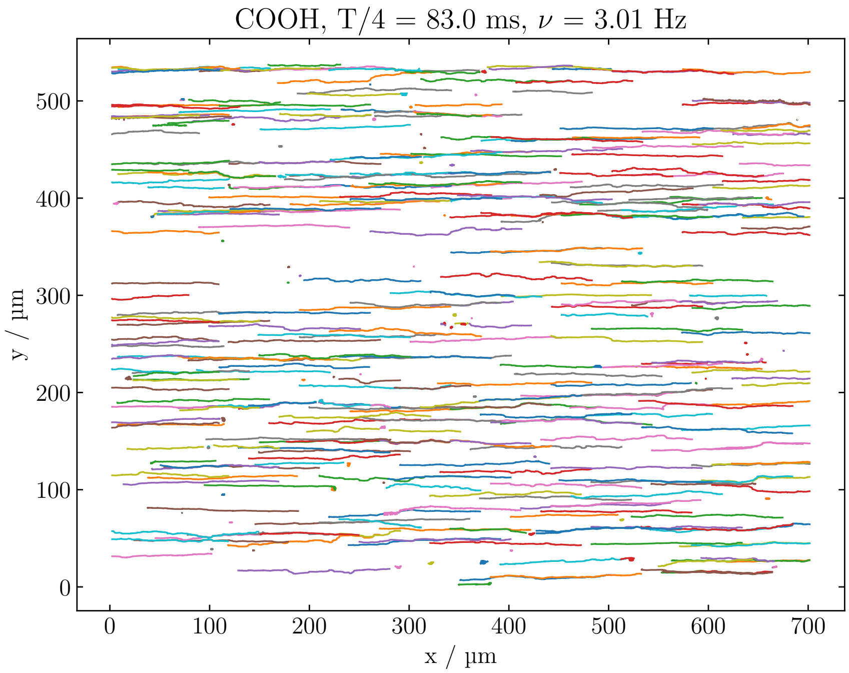

Figure S2: Trajectory map of COOH MPs at $\nu = 3.01\,\text{Hz}$, MPs present for less than 2000 frames removed.

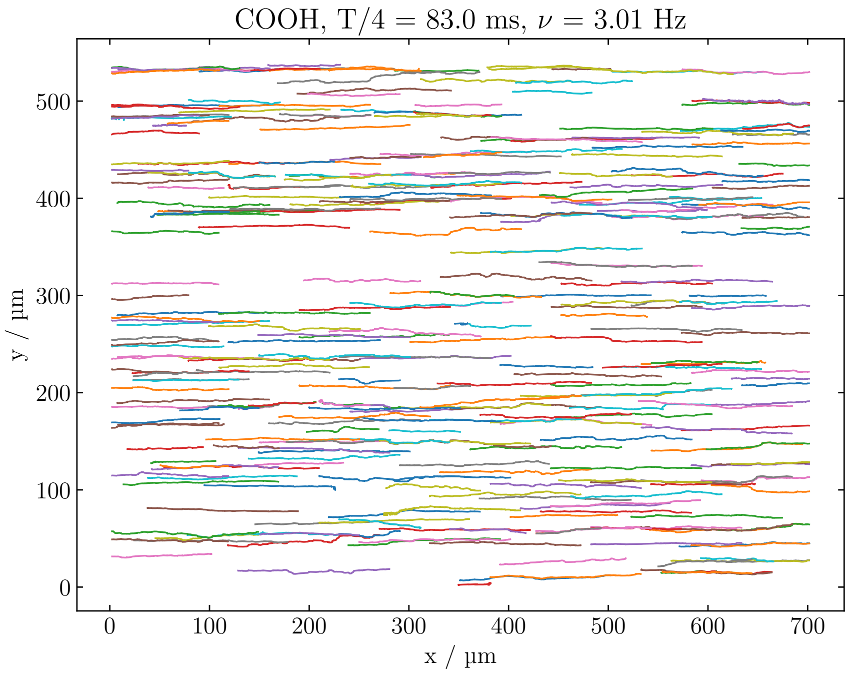

Figure S3: Trajectory map of COOH MPs at $\nu = 3.01\,\text{Hz}$, MPs present for less than 2000 frames and transported for less than 20 µm removed.

MP Trajectories, Number of Steps

Figure S5 depicts the lateral position x as a function of time t for one single MP that is transported at $\nu = 2.5\,\text{Hz}$ throughout the entirety of the experiment, which lasted 5 s. The zoom-in between 164 ms and 448 ms (red rectangle) is depicted in **Figure S6**. Two observations can be made: (1) The step-wise transport, where the small step is followed by a brief period of the MP being stationary, followed by a large step. (2) Within a given step, a brief acceleration is visible, followed by an (ideally) linear phase and a deceleration. While the small and big step sizes are identical for all frequencies, the total distance increases with the frequency ν within the linear regime as more steps are possible during a given time interval, i.e., a larger average velocity is reached. The number of either small or large steps is given by

$$n_{\text{steps}} = \frac{t}{\text{T}/2}. \tag{1}$$

As an example in Figure S5, an MP at $\nu = 2.5\,\text{Hz} \Rightarrow \text{T}/2 = 0.2\,\text{s}$ would be able to move 25 small and large steps. The phase relation between $\mathbf{H}_x$ and $\mathbf{H}_z$ is set to change after 40 steps, i.e., MPs perform 40 complete steps in the positive x-direction, then 40 steps back.

The inset of Figure 4 (a), where $\nu < \nu_{\text{c}}$, emphasizes that in addition to the lateral motion, MPs possess a small movement in the y-direction. This may occur due to one or several of the following origins: (1) Due to the sputtering process and the polycrystalline nature of the magnetic thin film, DWs will show slight inhomogeneity across the pattern, (2) The spin-coated polymer spacer presents height variations from the center outwards, (3) Brownian motion, lending the particles random-walk-like $y-$components, which is especially visible in **Figure S7** (b), (4) MPs colliding with defects on the substrate's surface, stuck MPs, or with MPs only performing oscillatory motion, and (5) slight mismatch between substrate- and x-field-orientation leading to DWs not perfectly perpendicular to the field direction, giving the MPs a magnetic force in y-direction. The latter would, however, lend all the MPs a preferable direction, which is not the case.

Figure S7 shows that given the high frequency of 25 Hz, 40 steps are performed quickly before the phase change between $\mathbf{H}_x$ and $\mathbf{H}_z$ changes the MP transport direction, meaning, 40 oscillations with an overall preferred direction (first $+x$) are followed by oscillations in the other direction (-x), well visible in the change of preferred direction in x. The overall directed transport is only possible because of the accumu-

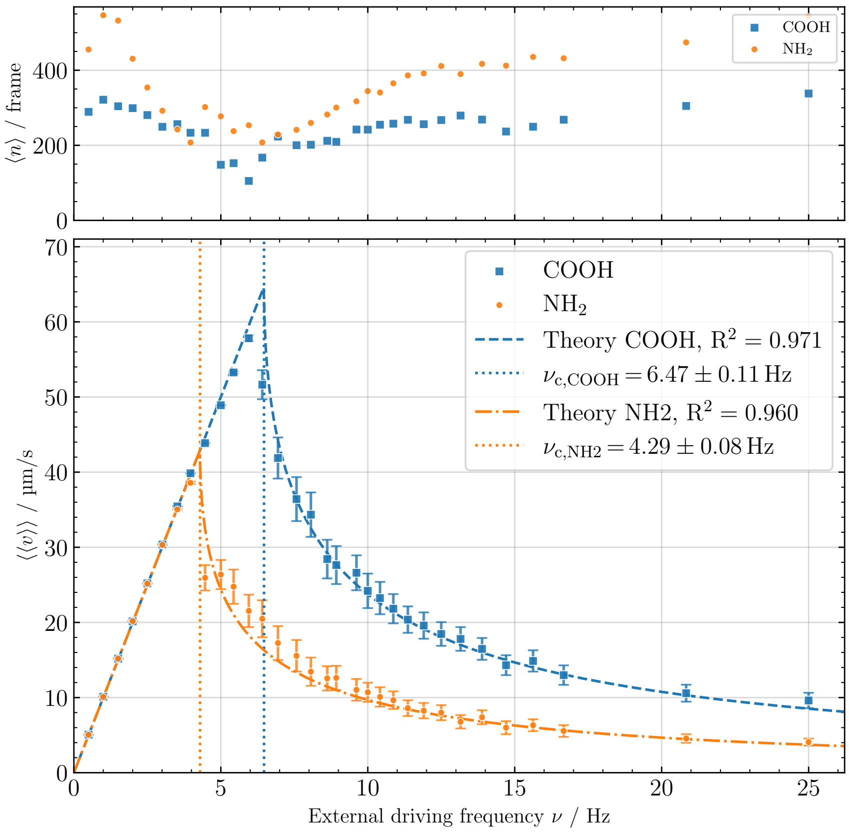

Figure S4: Average MP transport velocities $\langle\langle v \rangle\rangle\,(\nu)$ for the two differently surface-functionalized MP types averaged over the number of tracked MPs as a function of the external field pulse frequency ν. n MPs per ν is depicted in the top panel.

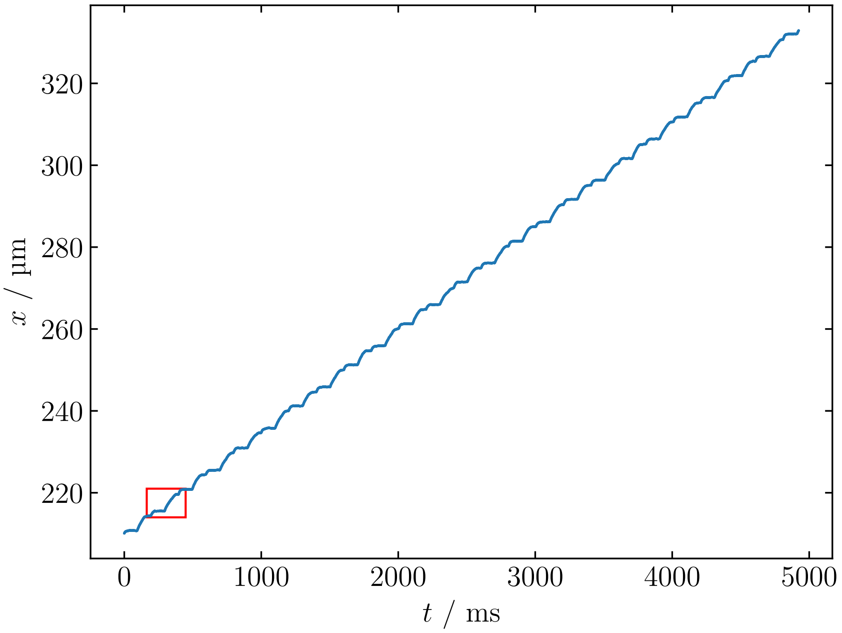

Figure S5: Trajectory of a single MP with COOH surface functionalization at a frequency of $\nu = 2.5\,\text{Hz}$. The red square indicates the area of interest visible in Figure S6.

lated slips back, where at each combined field pulse change in both $\mathbf{H}_x$ and $\mathbf{H}_z$, the MP is not able to follow from one DW to the next, but is constantly pushed along the direction, covering a fraction of the step that would be otherwise possible without slips-back at $\nu < \nu_c$. **Figure S8** shows the trajectory in the linear regime, hence, only few back slippages can be seen. This is in contrast to the oscillatory mo-

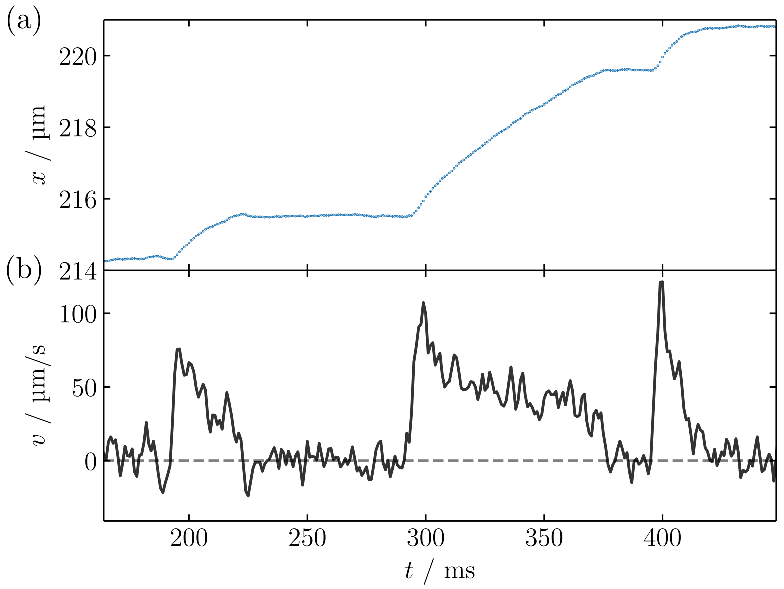

Figure S6: Cutout of stepwise transport in the linear regime at $\nu = 2.5$ Hz with its complete trajectory shown in Figure S5. (a) The trajectory shows a small step followed by a large step caused by the traveling wave magnetophoresis transport concept using pulsed external magnetic fields. One period corresponds to T/2 = 200 ms (see Figure 1.). (b) The time derivative of x gives the velocity.

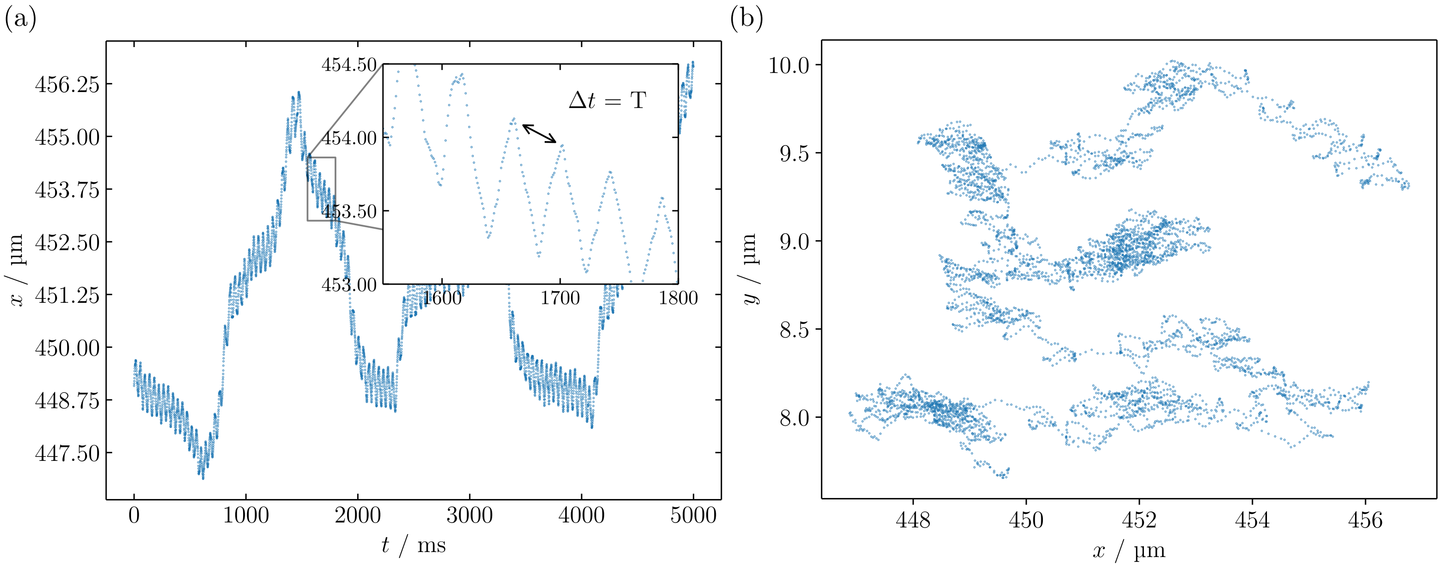

Figure S7: Trajectory of a single MP with COOH surface functionalization at a frequency of $\nu = 25$ Hz as a function of time t (a) and in the xy-plane (b) shows oscillatory motion around the domain walls. One period corresponds to T (Figure 1), which for $\nu = 25$ Hz is T = 10 ms. The directed transport alternates from negative x to positive x. Almost no effective directed transport is visible; the MP is transported a total of less than 40 µm within 5 s, while a transport distance of 1250 µm would be possible without back slippage.

tion shown in Figure S7. Here, one period is exactly T, making the 40 cycles visible (*small oscillation*) before the phase changes (*large oscillation*). An MP can not follow the moving potential minima, which leads to it falling back into the newly forming potential minima $\frac{\pi}{2}$ shifted. This would appear as oscillatory motion for higher driving frequencies visible in Figure S7. A slight motion along y, which is described above, is visible in Figure S7(b).

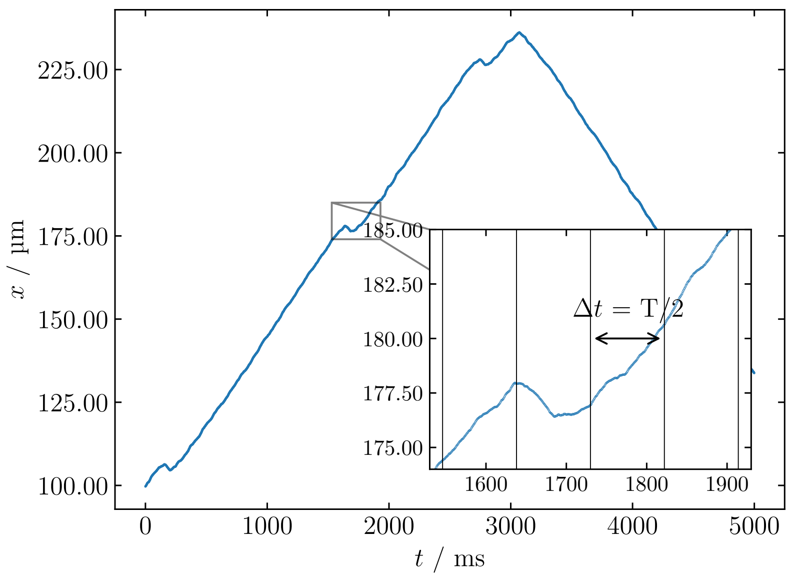

Figure S8: Trajectory of a single MP with NH_2 surface functionalization at a frequency of $\nu = 5.44\,\mathrm{Hz}$. Two features are recognized: (1) After around this frequency, individual parts of a step are no longer resolved (compare Figure S6), further indicated by the vertical black lines, where the acceleration of one large step should start, see Figure S6. (2) Back slippage is occurring, as $\nu > \nu_c$, here visible as small dips, where the MP falls back three times within 3000 ms.

Magnetophoretic Velocity, Far Away from any Surface

To ensure that the MPs used in this study have, indeed, the same magnetic content and are thus only distinguished due to the acting surface-specific DLVO forces close to the transport substrate, a magnetophoretic velocity study was conducted far away from any interacting surface (compare Figure 2). A suspension of MPs was placed in a 3D-printed container of 11 mm × 11 mm × 2 mm with a bar magnet attached to one wall, which can be seen in **Figure S9** (b). MPs were then attracted by the magnetic field gradient towards the magnet. The resulting mean magnetophoretic velocity $\langle\langle u \rangle\rangle$ derived from recorded videos was used to calculate the magnetic susceptibility χ_{MP} using[1]

$$\langle\langle u \rangle\rangle = \frac{V(\chi_{\mathrm{MP}} - \chi_{\mathrm{fl}})}{6\pi r \eta} \frac{|\nabla B^2|}{2\mu_0}, \tag{2}$$

with the magnetic flux density $B_{\mathrm{x}}(x)$ of the bar magnet used. $B_{\mathrm{x}}(x)$ in dependence of the distance to the magnet can be seen in Figure S9 (a). Videos with durations between 10 s and 50 s were recorded using a high-speed camera and dark-field microscopy with a 20 × magnification objective. The focus plane of the objective was set to about 1.5 mm below the coverslip in z, leaving 0.5 mm from the chamber surface as the closest surface, as the field-of-view was at least 2 mm away from any surface in x and y. A total of 442 trajectories for COOH and 155 trajectories for NH_2 functionalized particles were evaluated. At 88.0 mT, $\langle\langle u_{\mathrm{COOH}} \rangle\rangle = (149 \pm 16)\,\mu\mathrm{m\,s^{-1}}$ and $\langle\langle u_{\mathrm{NH2}} \rangle\rangle = (151 \pm 14)\,\mu\mathrm{m\,s^{-1}}$ were found. The resulting χ_{MP} are $\chi_{\mathrm{COOH}} = 0.26 \pm 0.01$ and $\chi_{\mathrm{NH_2}} = 0.26 \pm 0.01$, which is comparable to literature.[2]

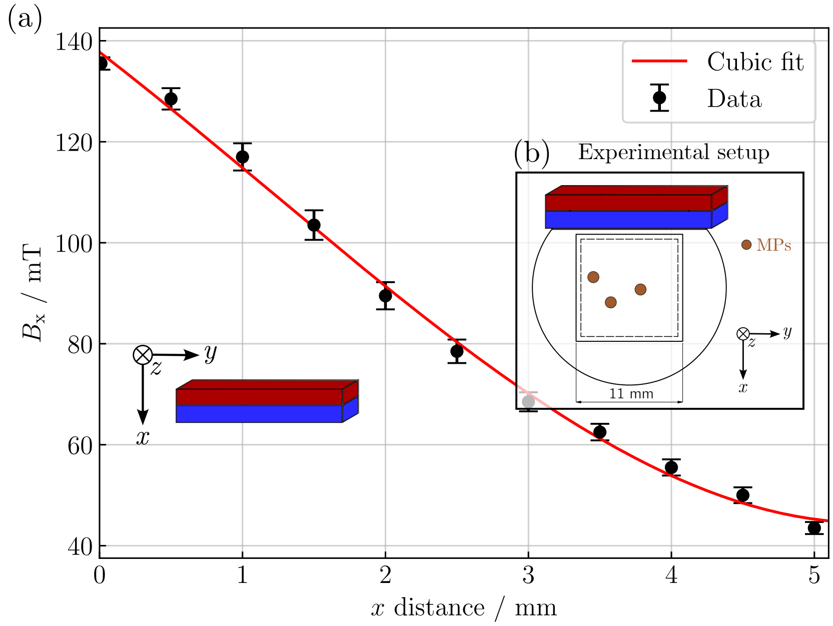

Figure S9: (a) Magnetic flux density in the investigated magnet orientation for different distances from the neodymium magnet attached to the wall at $x = 0$. (b) Top view of the experimental setup using a neodymium magnet with a 3D-printed fluid chamber sealed by a coverslip (not shown).

References

[1] N. Wise, T. Grob, K. Morten, I. Thompson, S. Sheard, Magnetophoretic velocities of superparamagnetic particles, agglomerates and complexes, *Journal of Magnetism and Magnetic Materials* **2015**, *384* 328, doi:10.1016/j.jmmm.2015.02.031.

[2] D. T. Grob, N. Wise, O. Oduwole, S. Sheard, Magnetic susceptibility characterisation of superparamagnetic microspheres, *Journal of Magnetism and Magnetic Materials* **2018**, *452* 134, doi:10.1016/j.jmmm.2017.12.007.
